# Leukocyte Derived Chemotaxin 2 (ALECT2) Amyloidosis

**DOI:** 10.4084/MJHID.2015.043

**Published:** 2015-07-01

**Authors:** Uday Kulkarni, Anna Valson, Anila Korula, Vikram Mathews

**Affiliations:** 1 Department of Clinical Haematology. Christian Medical College and Hospital, Vellore, Tamil Nadu, India; 2 Department of Nephrology. Christian Medical College and Hospital, Vellore, Tamil Nadu, India; 3 Department of General Pathology. Christian Medical College and Hospital, Vellore, Tamil Nadu, India

## Abstract

We describe the first case from India of ALECT2 amyloidosis. An adult Punjabi male presented with progressive renal dysfunction and non-nephrotic range proteinuria. Serum protein electrophoresis and immunofixation were normal, with mildly elevated serum free light chain ratio. A renal biopsy confirmed the presence of amyloid. Immunohistochemistry was negative for monoclonal light chains. Proteomic analysis confirmed the presence of ALECT2 amyloid. The present case highlights the need for confirmatory testing for typing of amyloid.

## Case

A 52 year old Punjabi male was evaluated elsewhere for bilateral leg swelling and reduced urine output. He was noted to have renal dysfunction and underwent a renal biopsy that was consistent with amyloidosis. Echocardiography revealed left ventricular diastolic dysfunction. Bone marrow revealed 10% plasma cells. Serum protein electrophoresis and immunofixation were normal. Free light chain ratio was mildly elevated (1.86). Whole body PET-CT (positron emission tomography-computed tomography) did not reveal any lytic lesions. He was diagnosed to have hypertension three years ago and was well controlled on oral cilnidipine. He was referred to our hospital as AL amyloidosis for autologous bone marrow transplantation.

At our hospital, general and systemic examination was unremarkable. His blood pressure was 130/80 mmHg. The renal biopsy was reviewed. It showed Congo red positive pale, amorphous, acellular, eosinophilic mesangial deposits consistent with amyloid. However, the immunohistochemistry was not conclusive. His creatinine was 1.47mg% and 24 hour urine protein was 99 mg. Urine Bence-Jones protein was negative. Serum protein electrophoresis and immunofixation were normal. Serum free light chain ratio was 4 (kappa 40mg/L; lambda 10mg/L). Review of the PET-CT did not reveal any lytic lesions. There was no anemia or hypercalcemia. Bone marrow was mildly hypercellular with nonspecific reactive changes. Echocardiography was normal.

**Figure 1:**
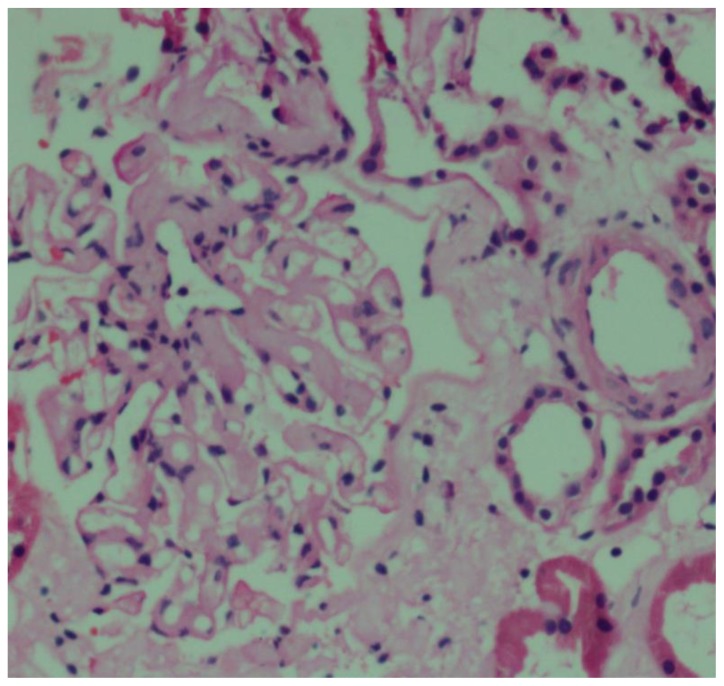
Glomerular, mesangial, capillary and interstitial amyloid deposits (hematoxylin and eosin staining × 200)

He underwent a repeat renal biopsy (Figure 1) which showed glomerular, megangial and capillary wall deposits of pale, acellular, eosinophilic material staining for amyloid with Congo Red and Thioflavine T histochemical stains. The interstitium and extraglomerular blood vessels showed similar deposits. Immunohistochemistry for monoclonal kappa and lamba light chains was negative. As the patient was referred to our hospital with a diagnosis of AL amyloidosis, the paraffin block was sent for proteomic analysis for exact typing of the amyloid. Liquid chromatography tandem mass spectrometry was performed at Mayo Clinic Laboratories, USA. The peptides were extracted from Congo Red positive microdissected paraffin embedded renal tissue. Mass spectrometry detected a peptide profile consistent with leucocyte-derived chemotaxin-2 type amyloidosis. Since there is no established treatment modality for ALECT2 amyloid, regular monitoring of renal function and blood pressure control were advised.

## Discussion

ALECT2, first reported in the year 2008, is one of the most recently described types of amyloidosis.^1^ It is the third most common type of systemic amyloidosis after AL and AA.^2^

LECT2 is a normal serum protein synthesized by the liver that is chemotactic for neutrophils, and a growth factor for chondrocytes and osteoblasts. However, the plasma levels of this protein are not increased in ALECT2 amyloidosis, and there are no identified mutations in *LECT2* causing misfolding leading to amyloidosis. A common *G/G* polymorphism at position 172 in *LECT2* leading to the replacement of isoleucine by valine may bring about a conformational change that predisposes to amyloidogenesis. However, a current theory suggests that it is a digenic disease, requiring a second mutation at an as yet unidentified locus.^3^

ALECT2 amyloidosis primarily involves the kidney, but liver, spleen, lung and adrenal involvement have also been described. It is common in certain ethnic groups like Hispanics, Arabs, and Punjabis.^4^,^5^ The usual presentation is of an elderly individual presenting with progressive renal insufficiency with or without proteinuria, which is usually non-nephrotic, unlike AL and AA amyloidosis, in which proteinuria is typically in the nephrotic range.^5^,^6^ Many of these patients also have a concomitant monoclonal gammopathy of unknown significance. Hence, it is important to distinguish these cases from AL amyloidosis to avoid unnecessary and often harmful chemotherapy.^3^

Although immunohistochemistry is a useful tool for confirming the type of amyloid, more accurate techniques like mass spectrometry based proteomic analysis are required in inconclusive cases.^7^ While anti-LECT2 antibody on immunohistochemistry can localize this form of amyloidosis to glomerular, interstitial and vascular deposits; liquid chromatography tandem mass spectrometry is used to confirm the diagnosis and exclude uncommon and familial forms of amyloid.

A concurrent renal disease like diabetic nephropathy and IgA nephropathy is common. Because cardiac involvement is rare, patient survival is superior to AL and AA amyloidosis. Median renal survival is 62 months in those without a concurrent renal disease. There is no available treatment for ALECT2 amyloidosis, except renal transplantation once end stage kidney disease is established.^8^

The present case highlights the following points:

1. Gradually progressing renal dysfunction even with a non-nephrotic range proteinuria may be a manifestation of renal amyloidosis.
2. When amyloid is noted histopathologically, the presence of a monoclonal gammopathy does not confirm the type of amyloid as AL.
3. The presence of predominantly renal interstitial and mesangial deposits of amyloid should alert one to the possibility of ALECT2 amyloidosis.
4. If immunohistochemistry is negative, mass spectrometry based proteomic analysis is necessary for confirming the type of amyloid.
5. ALECT2 amyloid may be an important cause of amyloid in India, especially among Punjabis.
6. It is important to establish this diagnosis to avoid chemotherapy or an autologous stem cell transplantation that may be considered in the absence of defining the type of amyloid.
